# Exploratory CSF proteomic analysis of a pre‐specified pTau217 subgroup from the SHINE clinical trial identifies biomarkers correlated with cognitive improvement in Alzheimer’s disease patients treated with zervimesine

**DOI:** 10.1002/alz70859_102120

**Published:** 2025-12-25

**Authors:** Britney N Lizama, Kiran Pandey, Eunah Cho, Jill Caldwell, Valentina Di Caro, Duc M Duong, Rick Shin, Allan I. Levey, Nicholas T Seyfried, Michael Grundman, Charlotte E. Teunissen, Anthony O Caggiano, Mary E. Hamby

**Affiliations:** ^1^ Cognition Therapeutics, Inc., Pittsburgh, PA USA; ^2^ Emory University School of Medicine, Atlanta, GA USA; ^3^ Global R&D Partners, LLC, La Jolla, CA USA; ^4^ University of California, San Diego, CA USA; ^5^ Neurochemistry Laboratory, Department of Laboratory Medicine, Amsterdam Neuroscience, Amsterdam UMC, Vrije Universiteit Amsterdam, Amsterdam Netherlands; ^6^ Cognition Therapeutics, Inc., Purchase, NY USA

## Abstract

**Background:**

Participants with Alzheimer’s disease (AD) treated with sigma‐2 receptor (S2R) modulator zervimesine (CT1812) exhibited slowing (39% in mITT, 95% in a pre‐specified plasma‐pTau217 subgroup (below‐median at baseline)) of cognitive decline (ADAS‐Cog11) compared to placebo in the SHINE trial (NCT03507790, COG0201). Given favorable clinical outcomes in the below‐median pTau217 subgroup, correlation analysis of the CSF proteome with ADAS‐Cog11 was performed to elucidate mechanisms of zervimesine‐mediated preservation of cognition.

**Method:**

SHINE was a Phase 2 randomized, double‐blind, placebo‐controlled study. Participants (N=152) received a daily oral dose of zervimesine (100 or 300 mg) or placebo for 6‐months (end‐of‐study). The median baseline plasma‐pTau217 concentration (ALZpath, SIMOA) designated participants into subgroups below median (<1 pg/mL, N=69) or above/equal median (≥1 pg/mL, N=69). A proteomics sub‐study of 45 participants was performed (tandem‐mass tag mass spectrometry, TMT‐MS) of baseline and 6‐month CSF. CSF from treatment‐compliant participants (N=43 mITT, N=17 below‐median) were used for further analyses. Pearson correlation analysis was performed with protein change‐from‐baseline (CFB) to ADAS‐Cog11 CFB. Pathway analyses (STRING, Metacore) were performed on correlated proteins (p≤0.01).

**Result:**

The previously‐reported mITT CSF cognitive correlates were compared to those in the below‐median pTau217 subgroup that demonstrated greater cognitive benefit with zervimesine versus placebo. In this subgroup, 106 proteins correlated with ADAS‐Cog11 (p≤0.01), exhibiting enrichment in amyloid biology and immune response pathways (p≤0.05). To elucidate the biological underpinnings of robust pharmacodynamic correlates of cognitive improvement, pathway analysis was performed on 62 proteins that correlated with ADAS‐Cog11 (p≤0.01) in both mITT and below‐median pTau217 subgroup (excluding correlates (p≤0.01) in the subgroup that did not exhibit cognitive benefit with zervimesine). These 62 proteins were enriched in immune response, complement, and synapse biology pathways (FDR≤0.05).

**Conclusion:**

In a pre‐specified subgroup exhibiting 95% slowing of cognitive impairment with zervimesine, we identified protein correlates of cognition related to amyloid biology, supporting zervimesine mechanism of action. Enriching the mITT analysis with correlates from this biomarker‐defined patient population reinforced observations of immune response and synaptic pathways that correlate with cognition in both mITT and below‐median pTau217 subgroup. These biomarker findings paired with positive clinical outcomes support the further clinical development of zervimesine for AD.

